# Supplementary material for: Turing instability in quantum activator–inhibitor systems
Source: Sci Rep. 2022 Sep 16;12:15573. doi: 10.1038/s41598-022-19010-0 (PMC9481611; doi:10.1038/s41598-022-19010-0)
Supplement: Supplementary file 1 — Supplementary Information 1. [file 41598_2022_19010_MOESM1_ESM.pdf]

Turing instability in quantum activator-inhibitor systems

- Supplementary Information -

Yuzuru Kato[1] and Hiroya Nakao[2]

[1] Department of Complex and Intelligent Systems, Future University Hakodate, Hokkaido 041-8655, Japan (Corresponding author: katoyuzu@fun.ac.jp)

[2] Department of Systems and Control Engineering, Tokyo Institute of Technology, Tokyo 152-8552, Japan

ABSTRACT

A Supplementary Figure and three Supplementary Movies are available.

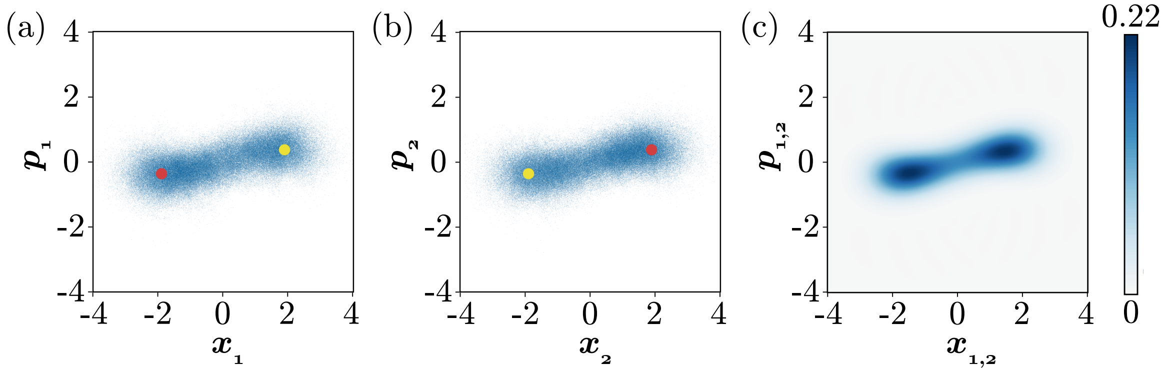

FIG. S 1. **Scatter plots of stochastic trajectories of two diffusively coupled quantum activator-inhibitor units described by Eq. (30) in the main text.** (a) (x_1, p_1) and (b) (x_2, p_2). The semiclassical SDEs of the two coupled units (a, b) have been numerically simulated up to $t = 4000$ with a time interval of $\Delta t = 0.02$ after the initial transient. (c) 2D density plot of the stationary Wigner distributions $W(x_1, p_1)$ and $W(x_2, p_2)$ of units 1 and 2, which are identical to each other. Red and yellow dots in (a, b) represent stable fixed points of the deterministic classical system. The parameters of quantum activator-inhibitor units are $\Delta = -0.6, \gamma_1 = 0.4, \gamma_2 = 0.1, \theta = \pi$, and $\eta = 0.3$ and the diffusion constants are $D_x = 0.005$ and $D_p = 0.995$ ($D_h = -0.99$ and $D_c = 1$).

Supplementary Movie S1:

Time evolution of the marginal Wigner distributions of the two units under continuous quantum measurement in the semiclassical regime in Fig. 7.

Supplementary Movie S2:

Time evolution of the marginal Wigner distributions of the two units under continuous quantum measurement in the weak quantum regime in Fig. 8.

Supplementary Movie S3:

Time evolution of the marginal Wigner distributions of the two units under continuous quantum measurement in the strong quantum regime in Fig. 9.
